# Association between CD10 expression and phyllodes tumor: A retrospective case control study

**DOI:** 10.1097/MD.0000000000035677

**Published:** 2023-11-03

**Authors:** Fang Tian, Jie Zhao, Qing-Fang Shi, Deng-Cai Zhang, Yue-Yuan Wang, Jian-Ping Sun, Yan Wang, Si-Ang Yang, Chong Zhang

**Affiliations:** a Pathology Department, Gansu Provincial Maternity and Child-care Hospital, Lan Zhou, China; b Gynecology Department, Gansu Provincial Maternity and Child-care Hospital, Lan Zhou, China; c Clinical Lab, Gansu Provincial Maternity and Child-care Hospital, Lan Zhou, China.

**Keywords:** breast neoplasms, fibroadenoma, Ki-67 antigen, phyllodes tumor, tumor suppressor protein p53

## Abstract

The present study aimed to explore the association between immunohistochemical markers and phyllodes tumor (PT). The retrospective case control study included biopsies from patients with PT who underwent surgical treatment, and patients with fibronenoma (FA), diagnosed in our hospital from October 2014 to May 2021. Differences in microscopic histopathological characteristics and expressions of common immunohistochemical markers (CD10, cluster of differentiation 117 marker, cluster of differentiation 34 marker, tumor protein P53, cell proliferation antigen) for different grades of PT and FA were analyzed. A total of 69 patients were enrolled, of them 34 with PT (12 with benign PT, 13 with borderline PT, and 9 with malignant PT) and 35 with FA. With the increase of tumor malignancy, significant enlargement trend was noted; for FA, most tumor boundaries were well-defined, the stromal distribution was homogeneous, the stromal cellularity was small. In contrast for PT, as the degree of malignancy increased, tumor boundary gradually became ill-defined and the stromal distribution was heterogeneous; stromal cellularity and stromal overgrowth had increased significantly (All *P < *.05). Multivariate analysis showed that among other markers only CD10 expression (OR = 0.67, 95%CI: −0.88, 2.22, *P* < .05) was independently associated with PT. The study showed that in addition to histological features, CD10 expression was independently associated with PT and has a potential to be used as a differentiation marker.

## 1. Introduction

Fibroepithelial tumors of the breast are heterogeneous group of true bidirectional lesions, comprising the fibroadenoma (FA) and phyllodes tumor (PT),^[1,2]^ with both epithelial and mesenchymal components. FA are the most common and may be treated conservatively,^[3]^ while PT are relatively rare lesions (0.3%–1% of primary breast tumors),^[2,4]^ classified as benign, borderline, or malignant based on various parameters. The median age of PT patients is relatively young, and malignancy prognosis is often challenging and imposes formidable demands on a pathologist skills.^[3]^

FA and PT have similar clinicopathologic manifestations and similar breast imaging features, but the clinical interventions and prognosis are quite different,^[1]^ so the accuracy of grading and differential diagnosis of PT has important clinical implications.^[4,5]^ Current World Health Organization (WHO) diagnostic criteria for breast tumor classification^[6]^ underline problems of high subjectivity and poor diagnostic repeatability, so seeking objective indices for grading and differential diagnosis of PT is still imperative.^[7]^

Diagnosis of PT is mainly based on pathological findings. Cellularity and cytologic atypia, fibroproliferative satellite nodules, positive margins, infiltrative borders, and past history of fibroadenoma are known to be morphologic predictors of local PT,^[8]^ while tumor size, necrosis, and stromal overgrowth can predict distant metastasis.^[5,9]^ However, these parameters are not definite markers of malignancy. Recent studies showed that immunohistochemical^[10]^ and molecular^[11,12]^ techniques have a great potential for differentiating FA from PT, but unlike other tumors, tissue availability, inter-observer variability, and heterogeneity make accurate evaluation of biopsies harder and those techniques yet to play a decisive role in the diagnosis, with limited reports available.

Thus, the study applied semi-quantitative immunohistochemical method to explore the association between immunohistochemical markers and PT.

## 2. Methods

### 2.1. Study design and participants

Patients who received surgical treatment and were pathologically diagnosed as lobular breast tumor in the department of breast Surgery of our hospital from October 2014 to May 2021 and patients with FA as the control group were retrospectively collected. Inclusion criteria: Cases of PT or FA diagnosed by 2 senior pathologists with double blind independent film reading and consistent results; Perfect preservation of clinical and pathological data. Exclusion criteria were as follows: Patients with other malignant tumors or severe underlying diseases; Lobular neoplasms without surgical treatment; Unable to cooperate with follow-up. The procedures followed were in accordance with the ethical standards of the responsible committee on human experimentation (institutional or regional) and with the Helsinki Declaration of 1975, as revised in 2000. The study was approved by the Ethics Committee of our hospital. Since this study was a retrospective study, the ethics Committee of the hospital approved the exemption of informed consent of the patients.

According to the 2018 WHO classification criteria for breast tumors,^[13]^ cases were divided into 3 subgroups: benign, borderline, and malignant, based on tumor boundary, stromal cellularity, stromal overgrowth, stromal atypia, and mitosis.

### 2.2. Data collection and definition

Various demographic and clinical data of patients were collected from the digital medical record system, including age, tumor volume, tumor pathological characteristics (tumor boundary, stromal cellularity, stromal uneven distribution, stromal atypia, stromal overgrowth, mitosis and phyllodes structure) and immunohistochemical data (tumor protein P53 [P53], cell proliferation antigen [Ki-67], cluster of differentiation 34 marker [CD34], cluster of differentiation 117 marker [CD117], and cluster of differentiation 10 marker [CD10]).

Tumor boundary was described as “well-defined” in the cases when there was an obvious pushing margin between the tumor tissue and the surrounding tissues. Stromal cellularity was described as “mild” if stromal cellularity was about twice that of normal lobular stromal cellularity, without nucleus overlap; as “severe” in cases of significant cell proliferation and nucleus overlap; as “moderate” in cases between mild and severe, with focal nucleus overlap. Stromal uneven distribution referred to the formation of a “sleeve-like” structure with abundant cells and surrounding epithelial components. Stromal atypia was described as “mild” if the cell outline was smooth, inconsistency in size was slight, and nuclear membrane was smooth; as “moderate” in obvious discernible cell size inconsistency and irregular nuclear membrane; as “severe” in cell size inconsistency, and markedly irregular nuclear membrane, chromatin thick and block-shaped, with nucleoli. Stromal overgrowth referred to the lack of epithelial components in at least one low-power field. Mitosis referred to the mitosis count of cell hot spot count/10 high-power field (about 0.5 mm in diameter), classified as 0 to 5, 6 to 11, and ≥12. Phyllodes structure referred to the stromal overgrowth protruding into the lumen to form a phyllodes structure.

### 2.3. Statistical analysis

The SPSS 19.0 software (IBM, Armonk, NY) was used for the statistical analysis of data. Continuous data with a normal distribution were presented as means ± standard deviation and tested using Student *t* test or analysis of variance; otherwise, they were presented as medians (interquartile range) and analyzed using Kruskal–Wallis test. Categorical data were expressed as n (%) and were analyzed using Fisher exact test. Logistic multivariate analysis was used to investigate the association between Ki-67, P53, CD34, CD117 and CD10 and PT. *P < *.05 was considered statistically significant.

## 3. Results

### 3.1. Patient characteristics

The present study enrolled 34 patients in the PT group and 35 FA patients in the control group. In the PT group, there were 12 with benign PT, 13 with borderline PT, and 9 with malignant PT. The difference in tumor volume in FA and each grade of PT was statistically significant, and showed a significant enlargement trend with the increase of tumor malignancy (*P < *.05); for FA, most tumor boundaries were well-defined; the stromal distribution was homogeneous, the stromal cellularity was small; there was basically no stromal overgrowth, and phyllodes structure was occasionally seen. In different grades of PT, as the degree of malignancy increases, the tumor boundary gradually became ill-defined and the stromal distribution was heterogeneous; stromal cellularity and stromal overgrowth had increased significantly, and the phyllodes structure was gradually more common. The development trend of the above indiced had a significant correlation with the increase in the degree of malignancy, and all *P < *.05; stromal atypia and mitosis were unrelated to the development trends of FA, benign, borderline, and malignant PT, with *P* values of 0.258 and 0.282, respectively. Among them, stromal atypia showed a significant increasing trend as the increase in malignancy in different grades of PT (*P < *.001); mitosis was also significant in differentiating FA and PT (*P < *.001). The specific results were shown in Table 1.

**Table 1:** Characteristics of patients.

Characteristic	FA (n = 35)	PT (n = 34)	*P*	PT (n = 34)			*P*
				Benign PT (n = 12)	Borderline PT (n = 13)	Malignant PT (n = 9)	
Age (yr), mean ± SD	43.2 ± 1.502	33.6 ± 1.418	<.0001	42.85	44.62	41.67	.7454
Tumor volume (cm^3^), median (IQR)	239.4 ± 77.22	10.76 ± 3.133	.0043	36.83	344	380.9	.1279
Tumor boundary, n (%)			<.0001				<.0001*
Well-defined	35 (100.0)	16 (47.06)		12 (100.0)	4 (30.77)	0	
Ill-defined	0	18 (52.94)		0	9 (69.23)	9 (100.0)	
Stromal cellularity, n (%)			<.0001*				<.0001*
Mild	35 (100.0)	12 (35.29)		10 (83.33)	1 (7.69)	1 (11.11)	
Moderate	0	14 (41.18)		2 (16.67)	12 (92.31)	0	
Severe	0	8 (23.53)		0	0	8 (88.89)	
Stromal distribution, n (%)			<.0001				.0891
Homogeneous	35 (100.0)	5 (14.71)		0	4 (30.77)	1 (11.11)	
Heterogeneous	0	29 (85.29)		12 (100.0)	9 (69.23)	8 (89.89)	
Stromal atypia, n (%)			<.0001*				<.0001*
None	35 (100.0)	2 (5.88)		2 (16.67)	0	0	
Mild	0	12 (35.29)		10 (83.33)	1 (7.69)	1 (11.11)	
Moderate	0	14 (41.18)		0	12 (92.31)	2 (22.22)	
Severe	0	6 (17.65)		0	0	6 (66.67)	
Stromal overgrowth, n (%)			.0002				<.0001*
None	35 (100.0)	23 (67.65)		12 (100.0)	11 (84.62)	0	
Yes	0	11 (32.35)		0	2 (15.38)	9 (100.0)	
Mitosis, n (%)			<.0001*				<.0001*
0–5	35 (100.0)	19 (55.88)		12 (100.0)	6 (46.15)	1 (11.11)	
6–11	0	11 (32.35)		0	7 (53.85)	4 (44.44)	
≥12	0	4 (11.76)		0	0	4 (44.44)	
Heterology of malignant cells, n (%)			.1531				.0001
None	100	97.06		100	100	88.89	
Yes	0	2.94		0	0	11.11	
P53, n (%)			<.0001*				.0808*
0–2	19 (54.29)	1 (2.94)		1 (8.33)	0	0	
3–4	0	3 (8.82)		3 (25.0)	0	0	
≥5	16 (45.71)	30 (88.24)		8 (66.67)	13 (100.0)	9 (100.0)	
Ki-67, n (%)			<.0001*				.0296
0–2	22 (62.86)	2 (5.88)		2 (16.67)	0	0	
3–4	0	3 (8.82)		3 (25.0)	0	0	
≥5	13 (37.14)	29 (85.29)		7 (58.33)	13 (100.0)	9 (100.0)	
CD10, n (%)			.0022				<.0001*
0–2	21 (60.0)	8 (23.53)		8 (66.67)	0	0	
≥5	14 (40.0)	26 (76.47)		4 (33.33)	13 (100.0)	9 (100.0)	
CD117, n (%)			.0023*				.7042*
0–2	0	8 (23.53)		2 (16.67)	4 (30.77)	2 (22.22)	
≥5	35 (100.0)	26 (76.47)		10 (83.33)	9 (69.23)	7 (77.78)	
CD34, n (%)			.2436*				.1142*
0–2	4 (11.43)	6 (17.65)		0	3 (23.08)	3 (33.33)	
3–4	0	2 (5.88)		2 (16.67)	0	0	
≥5	31 (88.57)	26 (76.47)		10 (83.33)	10 (76.92)	6 (66.67)	

CD10 = cluster of differentiation 10 marker, CD17 = cluster of differentiation 117 marker, CD34 = cluster of differentiation 34 marker, FA = fibronenoma, Ki-67 = cell proliferation antigen, P53 = tumor protein P53, PT = phyllodes tumor.

* Represents the use of Fisher exact test.

†represents the use of Kruskal–Wallis test.

The present study analyzed immunohistochemical indices with potential clinical significance: p53, Ki-67, CD34, CD117, and CD10 in differential and grading diagnosis of PT. The cross tab Somers’d correlation analysis showed that, with the development of FA, benign, borderline, and malignant PT, the protein expressions of P53, Ki-67, and CD117 showed a significant increase trend (all *P < *.05); although the expression of CD10 was increased with the increase in the malignant degree of fibroepithelial tumors, there was no statistically significant difference (*P > *.05); the expression of CD34 showed a significant weakening trend with the development of FA, benign, borderline, and malignant PT (*P < *.05), and the specific results were shown in Table 1.

### 3.2. Analysis of influencing factors for PT

The expression of P53 (OR = 3.57, 95%CI 1.47, 5.67; *P* < .001), Ki-67 (OR = 3.20, 95%CI 1.61, 4.79; *P* < .001) and CD10 (OR = 1.58, 95%CI 0.54, 2.63; *P* < .01) were associated with PT in univariate analysis, while expression of CD117 and CD34 showed no significant difference between PT and FA. Multivariate analysis showed that only CD10 expression (OR = 0.67, 95%CI: −0.88, 2.22, *P* < .05) was independent risk factor for PT development (Table 2).

**Table 2:** Univariate and multivariate analyses of risk factors for Phyllodes Tumor.

Variables	Univariable analysis		Multivariable analysis	
	OR (95%CI)	P	OR (95%CI)	*P*
P53				
0–2	Reference	/	Reference	/
3–4	19.51 (2695.78, 2734.80)	>0.05	57.04 (−58473.78, 58587.86)	>.05
≥5	3.57 (1.47, 5.67)	<0.001	35.43 (−11930.95, 12001.81)	>.05
Ki-67				
0–2	Reference	/	Reference	/
3–4	19.96 (−4456.79, 4496.72)	>0.05	23.15 (−29829.53, 29875.82)	>.05
≥5	3.20 (1.61, 4.79)	<0.001	2.15 (−0.18, 4.48)	>.05
CD10				
0–2	Reference	/	Reference	/
≥5	1.58 (0.54, 2.63)	<0.01	0.67 (−0.88, 2.22)	<.05
CD117				
0–2	Reference	/	Reference	/
≥5	−17.86 (−2759.31, 2723.58)	>0.05	−53.57 (−18587.55, 18480.41)	>.05
CD34				
0–2	Reference	/	Reference	/
3–4	16.16 (−3309.38, 3341.70)	>0.05	−20.18 (−67149.01, 67108.45)	>.05
≥5	−0.58 (−1.95, 0.79)	>0.05	−18.46 (−8338.25, 8301.33)	>.05

CD10 = cluster of differentiation 10 marker, CD17 = cluster of differentiation 117 marker, CD34 = cluster of differentiation 34 marker, Ki-67 = cell proliferation antigen, P53 = tumor protein P53.

## 4. Discussion

The study found that most tumor boundaries were well-defined, stromal distribution was homogeneous, the stromal cellularity was small for FA. In contrast for PT, as the degree of malignancy increased, tumor boundaries gradually became ill-defined and the stromal distribution was heterogeneous; stromal cellularity and stromal overgrowth had increased significantly. Multivariate analysis showed that only CD10 expression was independently associated with PT. Obtained results might help in grading diagnosis of PT and differential diagnosis of FA.

Due to the high heterogeneity of PT, its accurate pathological grading diagnosis is crucial for the selection of clinical interventions.^[14]^ At present, the differential and grading diagnosis of PT are mainly performed according to the criteria of the WHO 2012 or 2018 edition, but there are problems of high subjectivity and poor repeatability, so the reliability is still limited in providing the pathological basis for the accurate clinical diagnosis and intervention of PT. Present study performed semi-quantitative retrospective analysis of 34 breast PT cases and 35 age-matched FA ones. The histopathological features such as tumor boundary, stromal cellularity, stromal distribution, and overgrowth, as well as phyllodes structure overlapped between FA and PT, but as the nature of the tumor developed from benign to borderline, and malignant PT, there was a significant difference in features: in terms of stromal atypia there was a significant progression with the increase of tumor grade, and the correlation was statistically significant. The above histopathological characteristics are reflected in some other literature reports,^[15–17]^ however our results do not confirm significance of mitosis, stressed by some authors. In our study mitosis demonstrated histological overlap between FA and PT and although there was a certain progression with the development of the tumor, correlation was not significant. This mainly manifested as several FA and benign PT sometimes had a certain amount of mitosis, so the effect in differentiating between FA and benign PT was poor. Given all indices except for stromal atypia show overlap between FA and PT, comprehensive evaluation in the differential and grading diagnosis of PT is particularly important. In order to avoid the problems of high subjectivity and poor repeatability, semi-quantitation of each index can be a good preventive measure.

Immunohistochemistry technique has always played a crucial role in pathological diagnosis, especially differential diagnosis of tumors. However, immunohistochemical indices are not included in the WHO 2012 edition of breast epithelial tumor classification criteria. The reason for that is the low incidence of PT and fewer evidence-based studies. In the present study, some commonly used indices mentioned in other studies^[18]^ were included in the differential and grading diagnosis of PT. The results showed that the expressions of P53, Ki-67, and CD117 were increased with the increase of tumor malignancy. CD34 had a trend of decreased expression with the increase of tumor malignancy, and the correlation was statistically significant; CD10 had no statistical significance in the differential and grading diagnosis of PT. It is mainly consistent with the previous study by Liu et al,^[19]^ who reported similar changes for CD34, CD10, and CD117 in the pathological diagnosis of breast phyllodes tumors and other individual reports,^[20,21]^ suggesting that although various previously reported immunohistochemical indices overlapped in FA and different grades of PT, none of the indices had a unique diagnostic value. However, expression of P53 and Ki-67 that was associated with PT in univariate analysis in our study, have a potential to be more objective and repeatable. Previously Ki-67 expression was successfully used for reclassification of luminal breast cancer,^[22]^ while p53 is the main regulator of genomic stability and its overexpression is the most frequent genetic alteration in breast cancer.^[23]^ Combination of p53 and Ki-67 expression was reported to improve the prognostic power in other types of breast cancer^[24]^ and with the addition of CD10 has the potential to provide the auxiliary significance for histological diagnosis of PT.

This study also has some limitations. Firstly, due to the retrospective nature of the study, only cases with surgical biopsies were included. In addition, data related to the prognosis was not available; in the future prospective study might further explore the relationship between CD10 and patient prognosis. Secondly, it was a single center study and although results were confirmed by 2 blinded pathologists, some bias in qualitative characterization might still exist. Finally, expression of all markers was evaluated by semi-quantitative method, without usage of any counting software. This approach shortens the time of analysis, making it more affordable, but inevitably leads to decreased precision and reproducibility of the results.

The study showed that only CD10 expression was independent risk factor of PT. It might help in grading diagnosis of PT and differential diagnosis of FA.

## Author contributions

**Conceptualization:** Chong Zhang.

**Data curation:** Fang Tian, Jie Zhao, Deng-Cai Zhang, Yue-Yuan Wang, Jian-Ping Sun, Yan Wang.

**Investigation:** Fang Tian, Jie Zhao, Qing-Fang Shi, Si-Ang Yang.

**Methodology:** Chong Zhang.

**Writing – original draft:** Fang Tian, Jie Zhao, Qing-Fang Shi, Deng-Cai Zhang, Yue-Yuan Wang, Jian-Ping Sun, Yan Wang, Si-Ang Yang, Chong Zhang.

**Writing – review & editing:** Fang Tian, Jie Zhao, Qing-Fang Shi, Deng-Cai Zhang, Yue-Yuan Wang, Jian-Ping Sun, Yan Wang, Si-Ang Yang, Chong Zhang.
